# Default Mode Network Functional Connectivity: A Promising Biomarker for Diagnosing Minimal Hepatic Encephalopathy: CONSORT-Compliant Article

**DOI:** 10.1097/MD.0000000000000227

**Published:** 2014-12-12

**Authors:** Rongfeng Qi, Long Jiang Zhang, Song Luo, Jun Ke, Xiang Kong, Qiang Xu, Chang Liu, Heng Lu, Guang Ming Lu

**Affiliations:** From the Department of Medical Imaging, Jinling Hospital, Clinical School of Medical College, Nanjing University, Nanjing, Jiangsu, 210002, China (RQ, LJZ, SL, JK, XK, QX, GML); Department of Gastroenterology, Jinling Hospital, Clinical School of Medical College, Nanjing University, Nanjing, Jiangsu, 210002, China (CL, HL).

## Abstract

To investigate the contribution of brain default mode network (DMN) in the early diagnosis of the minimal hepatic encephalopathy (MHE), the mildest form of HE from cirrhotic patients by using resting-state functional magnetic resonance imaging (rs-fMRI).

This study was approved by the local ethical committee, and a written informed consent was obtained from each participant. A total of 103 cirrhotic patients (34 MHE, 69 non-HE) and 103 matched healthy controls underwent rs-fMRI scanning. The DMN correlation map was acquired by using unbiased seed-based functional connectivity analysis and compared among MHE patients, non-HE patients, and healthy controls with analysis of variance tests. Pearson correlation analysis was performed between the abnormal DMN connectivity and neuropsychological performances. Receiver operator characteristic (ROC) analysis was used to evaluate the contribution of DMN connectivity strength in the differential diagnosis between MHE and non-HE.

Compared with the healthy controls, MHE and non-HE patients showed decreased DMN connectivity in medial prefrontal cortex (MPFC), left superior frontal gyrus (SFG), left temporal lobe, and bilateral middle temporal gyri (MTG). The MHE patients showed even more decreased connectivity in MPFC, left SFG, and right MTG when compared with non-HE patients. Pearson correlation analyses revealed that the decreased connectivity strength of some DMN regions correlated with patients’ neuropsychological tests scores. Connectivity strength of the MPFC, right MTG, and left SFG could differentiate MHE from non-HE, of which the MPFC had the highest effectiveness (sensitivity = 81.5%, specificity = 70.4%).

Cirrhotic patients had gradually reduced DMN functional connectivty from non-HE patients to MHE patients. DMN function, especially the MPFC, might be a useful imaging marker for differentiating MHE from cirrhotic patients.

## INTRODUCTION

Minimal hepatic encephalopathy (MHE) is a complication of liver cirrhosis that is characterized by the presence of cognitive alterations undiagnosed by routine clinical examination and identified solely through psychometric or neurological tests.^1^ MHE exists with high prevalence in cirrhotic patients (30%–84%).^2,3^ It is suggested that MHE patients often have markedly reduced health-related quality of life, impaired ability to work, increased risk of fall and traffic accidents, as well as poor survival.^4,5^

Therefore, the early diagnosis of MHE has great clinical importance.^6^ However, unlike the overt HE patients who had obviously neuropsychiatric symptoms, MHE is not detectable by routine clinical examinations, it is hard to distinguish the MHE from patients without any signs of HE (non-HE) in clinical practice.^7^ The diagnostic criteria for MHE have not been standardized until now, although some specific neuropsychological/neurophysiological tests are recommended, in which the neuropsychological tests are mostly used in clinical practice.^7–9^ However, the neuropsychological tests have some disadvantages, including the occurrence of learning effects, which limit their repeatability, and many confounding factors such as age, training, education and eyesight, which limits their availability.^1^ Thus, it is urgent to develop objective and quantitative methods for the early diagnosis of MHE.

In the past several years, resting-state functional magnetic resonance imaging (rs-fMRI) has been wildly used as an important technique in uncovering the neuropathological mechanism of HE.^10–12^ Several rs-fMRI studies in HE have been performed with a focus on the brain default mode network (DMN),^13–15^ which is unique in terms of the higher activity of the idling brain. Zhang et al^15^ first showed a reduction of DMN functional connectivity by using rs-fMRI in a group of overt HE. Qi et al^14^ found that MHE patients had DMN functional alterations preceding its structural alterations. Furthermore, there are some evidences that DMN function is helpful in differential diagnosis of brain disorders, including cognitive difficulties caused by cancer chemotherapy,^16^ Alzheimer's disease,^17^ and even the mild cognitive impairment.^18,19^ Compared with other neuroimaging techniques that ever been used in patients with cirrhosis, rs-fMIR has the advantage of no radiation exposure (compared to position emission tomography [PET] and single photon emission computed tomography [SPECT]), easy application (compared to task-driven paradigms), and good spatial resolution (compared to PET and SPECT, and MR spectroscopy).

In the present rs-fMRI study, we hypothesized that DMN function could contribute to the early diagnosis of MHE. To test our hypothesis, an unbiased seed-based functional connectivity analysis of DMN was compared among MHE patients, non-HE patients, and healthy controls, and the receiver operator characteristic (ROC) measurement was performed to evaluate the effectiveness of DMN in differentiating the MHE from non-HE.

## MATERIALS AND METHODS

### Subjects

This study protocol was approved by the Medical Research Ethics Committee of Jinling Hospital. Written informed consents were obtained from all participants prior to the study between June 2009 and January 2014.

A total of 103 cirrhotic patients without clinical signs of HE (81 men, 22 women, and mean age: 48.0 ± 10.3 years) were recruited from our inpatient and outpatient departments. The inclusion criteria for recruitment of the patients were as follows: patients with clinically proven hepatic cirrhosis, without clinical manifestation of HE, age 18 years or older, who could finish the MR exam without any MRI contraindication. Exclusion criteria for all the subjects included any drug abuse history, and any obvious brain lesions such as tumor or stroke assessed on the basis of medical history and conventional MRI. Subjects who had motions with translation more than 1.0 mm or rotation more than 1.0° during MRI were also excluded from further fMRI data analysis.

The diagnosis of MHE was made according to a final report of the working party of 11th World Congress of Gastroenterology in Vienna in 1998.^8^ Two typical neuropsychological tests, number connection type-A (NCT-A) and digit symbol test (DST) were performed on all subjects before MRI scanning. When the scores of at least one test were abnormal, the cirrhosis patients would be regarded as MHE.^20,21^ According to this criterion, 34 of all the 103 patients with cirrhosis (33.0%) were diagnosed to have MHE. In addition, 103 age- and gender-matched healthy controls (72 men, 31 women, mean age: 47.4 ± 10.1 years) were recruited from the local community by means of poster advertisement between June 2009 and January 2014. All healthy controls had no diseases of the liver (cirrhosis, hepatitis, liver tumors, or extrahepatic portal vein obstruction) and other systems. All these healthy controls underwent neuropsychological tests before the MR imaging.

### Laboratory Examinations

Laboratory parameters including prothrombin time, protein metabolism tests, and venous blood ammonia were obtained during the week before MRI from all patients to assess the severity of liver disease. The grade of hepatic function was determined according to the Child-Pugh score.^22^ Of these 103 patients, 58 patients had Child-Pugh grade A, 42 patients had Child-Pugh grade B, and 3 patients had Child-Pugh grade C. No laboratory tests were performed, thus unavailable for healthy controls.

### MRI Data Acquisition

Patients and healthy controls underwent scanning using a 3 Tesla MR scanner (TIM Trio, Siemens Medical Solutions, Erlangen, Germany). Foam padding was used to minimize the head motion for all participants. Resting-state functional images were obtained using a single-shot, gradient-recalled echo planar imaging sequence (250 volumes, TR/TE = 2000 ms/30 ms, FOV = 240 mm × 240 mm, flip angle = 90°, matrix = 64 × 64, voxel size = 3.75 mm × 3.75 mm × 4 mm, 30 axial slices aligned along the anterior-posterior commissure).

### Data Preprocessing

Preprocessing of functional images was carried out using SPM8 software package (http://www.fil.ion.ucl.ac.uk/spm). The first 10 volumes were excluded to ensure steady-state longitudinal magnetization, and the remaining images were corrected for temporal differences and head motion. No translation or rotation parameters in any given data set exceeded 1.0 mm or 1.0° and there were no group differences for both head motion parameters (two-sample *t* test, all *P* > 0.05 for translational and rotational motion). Then the functional images were spatially normalized to the Montreal Neurological Institute (MNI) template (3 × 3 × 3 mm^3^) by applying a 12-parameter affine transformation, followed by a nonlinear warping using basis functions.^23^ Images were then smoothed by convolution with an isotropic Gaussian kernel of 8 mm FWHW to decrease spatial noise. In order to further reduce the effects of confounding factors unlikely to be involved in specific regional correlation, several sources of spurious variance by linear regression, including six head motion parameters, and average signals from cerebrospinal fluid, white matter, and whole brain, are also removed.^24^ Then, the residual time series were band-pass filtered (0.01–0.08 Hz) using the REST1.8 software (http://resting-fmri.sourceforge.net).

### Seed-based Functional Connectivity of DMN

According to previous fMRI studies,^24,25^ the medial prefrontal cortex (MPFC) (MNI coordinates: −1, 47, −4), the posterior cingulate cortex (PCC) (MNI coordinates: −5, −49, 40), and left lateral parietal cortex (LP) (MNI coordinates: −45,−67, 36) were selected for detecting the DMN. A temporal correlation map was conducted by computing the cross-correlation coefficient (*r* score) between the each seed region and each voxel within the whole brain. Correlation coefficients were then converted to *z* values using Fisher's *r*-to-*z* transform to standardize the statistical analysis. The significance level was set at *P* < 0.05.

### Conjunction Analysis

The unbiased DMN was mapped by conjunction analysis^24,25^ of the 3 *z* maps with similar spatial patterns of each network. The voxels whose functional connectivity survived at a threshold at *P* < 0.05 were corrected for multiple comparisons using false discovery rate (FDR) criterion. The average was then masked by using a conservative conjunction procedure. Voxels were included in the mask only if they were significantly correlated with at least 2 of the 3 seed regions.^26^

### Statistical Analysis

Within each group, a random-effect one-sample *t* test was performed in individual DMN map after conjunction. Significant thresholds were set at a corrected *P* < 0.05, using FDR criterion.

To examine the difference among 3 groups, one-way analysis of variance (ANOVA) was performed to determine the DMN differences among MHE, non-HE, and healthy control groups, and post hoc *t* tests were then performed to further examine the difference between groups within the significant regions detected by ANOVA, age, and gender were importing as covariates. Statistical threshold was also set at *P* < 0.05, corrected by using the AlphaSim program.

To investigate the relationship between neuropsychological performance and DMN functional connectivity in patients, the mean *z-*values of the DMN regions that differed significantly among three groups (ANOVA result) were correlated against the scores of NCT-A and DST of all the patients with the Pearson correlation analysis, using SPSS 16.0 (SPSS Inc., Chicago, IL), the threshold was set at a significance level of *P* < 0.05. Receiver operator characteristic (ROC) measurements were used to evaluate the ability of functional connectivity strength of DMN regions to distinguish the MHE from the non-HE patients. Regions from the ANOVA results were selected as region of interest (ROI) for ROC analysis, the threshold was set at a significant level of *P* < 0.05.

## RESULTS

### Demographics and Clinical Data

In the present study, the prevalence of MHE in all patients with cirrhosis was 33.0% (34/103). Demographics and clinical data for all the 206 participants were summarized in Table 1. All subjects were right-handed. There were no significant differences in gender, age, and years of education between patients and healthy control groups (all *P* > 0.05). However, patients demonstrated worse neuropsychological performance than healthy controls (both *P* < 0.05); in detail, they spent more time to complete the NCT-A, and had lower scores of DST (Table 1).

**TABLE 1:**
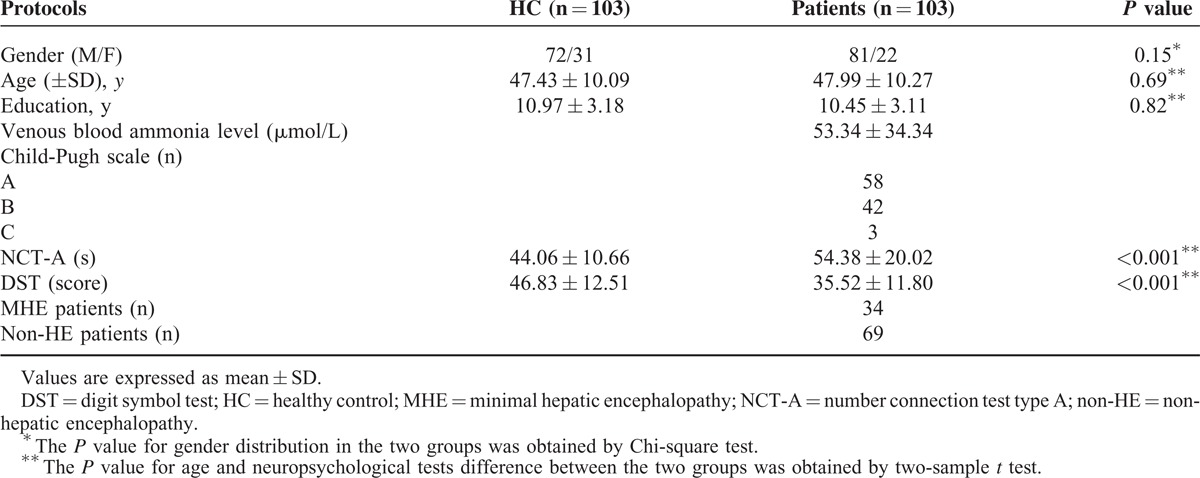
Demographics and Clinical Data of All Cirrhotic Patients and Healthy Controls

Functional MR data from one MHE patient and one healthy control were excluded because of excessive movement. Thus, 33 MHE patients, 69 non-HE patients, and 102 healthy control subjects were included in the final data analysis.

### Within-Group Results of DMN Conjunction Map

**FIGURE 1:**
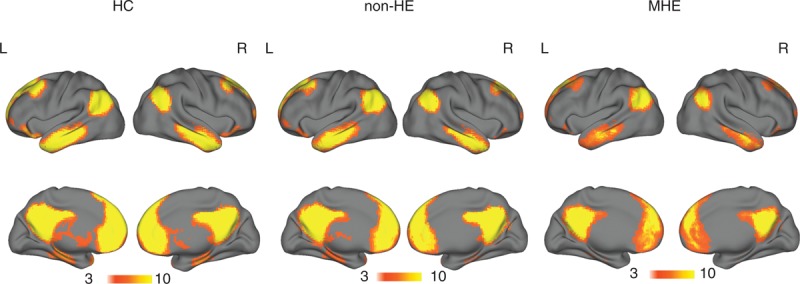
The main pattern of DMN correlation maps in healthy controls, non-HE, and MHE patients. Within each group, the DMN mainly includes the MPFC, SFG, PCC/precuneus, IPL, ITG, MTG, and PHG. DMN = default mode network; IPL = inferior parietal cortices; ITG = inferior temporal gyrus; MHE = minimal hepatic encephalopathy; MPFC = medial prefrontal cortex; MTG = middle temporal gyrus; non-HE = non-hepatic encephalopathy; PCC = posterior cingulate cortex; PHG = parahippocampal gyrus; SFG = superior frontal gyrus.

Within each group, the main spatial pattern of DMN correlation map was similar by visual inspection (Figure 1). The DMN mainly included the MPFC, PCC/precuneus, bilateral inferior parietal cortices (IPL), inferior/middle temporal cortices, parahippocampal gyri, and superior frontal gyri (SFG), highly consistent with previous studies.^24,25^

### Group Differences of DMN Functional Connectivity

**FIGURE 2:**
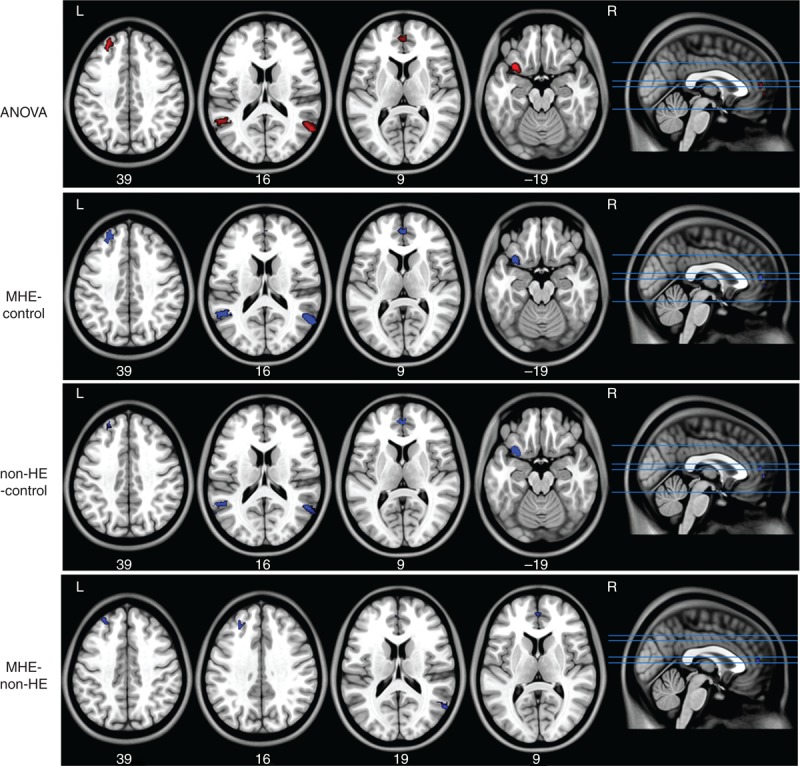
Group differences of DMN functional connectivity. Compared with the healthy controls, a significantly decreased DMN functional connectivity is found in non-HE patients and MHE patients in MPFC, left SFG, ITG, MTG, and left temporal lobe. MHE patients also show decreased connectivity in MPFC, left SFG, and right MTG when compared with non-HE patients. ANOVA = analysis of variance; DMN = default mode network; ITG = inferior temporal gyrus; MHE = minimal hepatic encephalopathy; MPFC = medial prefrontal cortex; MTG = middle temporal gyrus; non-HE = non-hepatic encephalopathy; SFG = superior frontal gyrus.

ANOVA result showed significant difference of DMN functional connectivity in 5 regions: MPFC, left SFG, left temporal lobe, and bilateral MTG. Compared with the healthy controls, both the MHE and non-HE patients showed decreased DMN connectivity in MPFC, left SFG and temporal lobe, and bilateral MTG. Compared with the non-HE patients, MHE patients showed even more decreased DMN connectivity in MPFC, left SFG, and right MTG (Figure 2, Table 2).

**TABLE 2:**
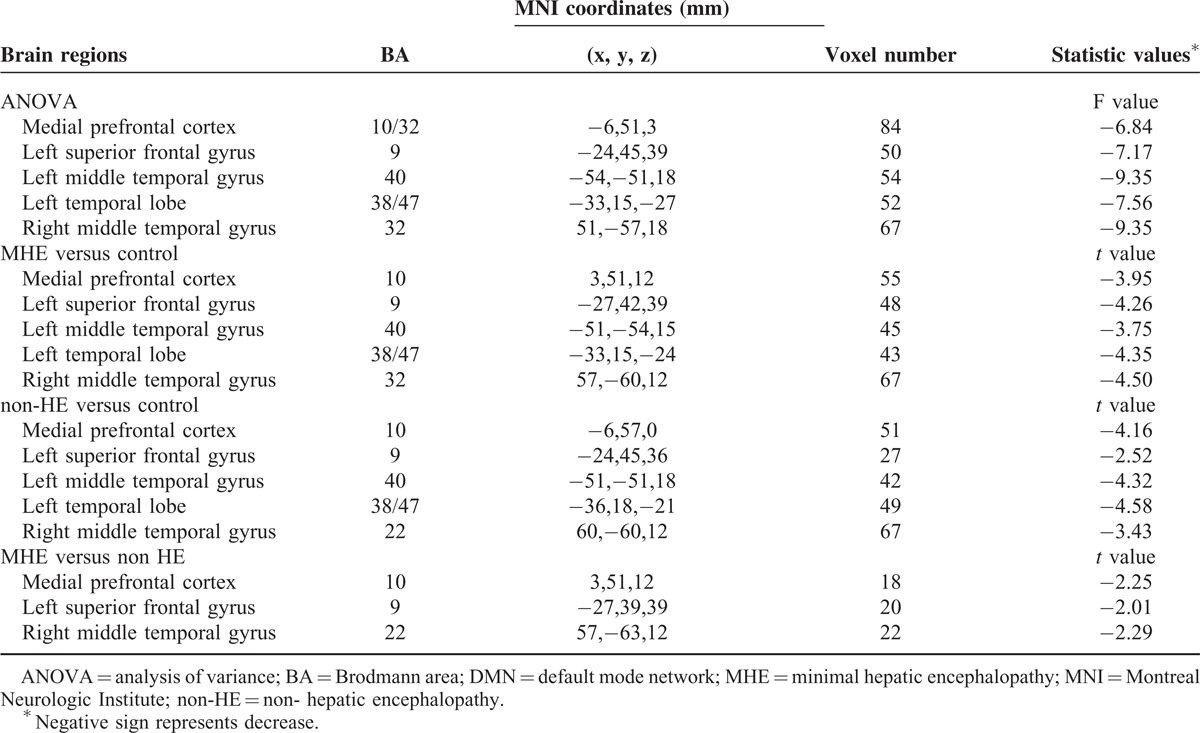
Regions Showing DMN Functional Connectivity Differences

### Correlations Results

**FIGURE 3:**
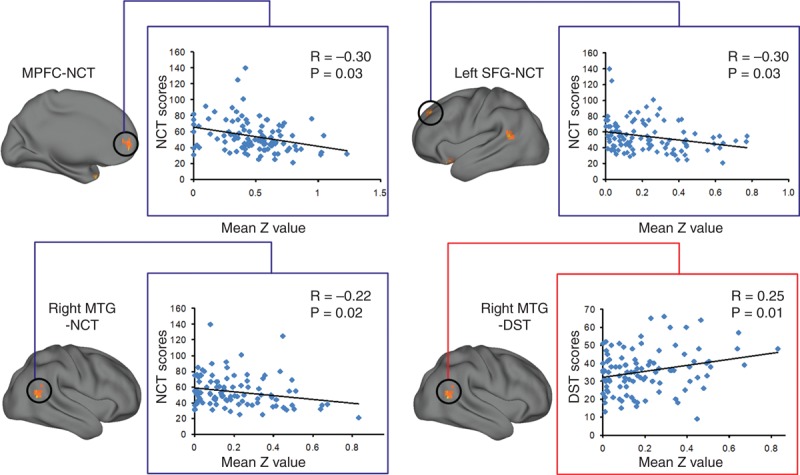
Correlation results between abnormal DMN functional connectivity and neuropsychological performance. Pearson correlation analyses reveal that the NCT scores of all cirrhotic patients negatively correlate with DMN functional connectivity in the MPFC, left SFG, and right MTG. DST scores show positive correlation with DMN functional connectivity in the right MTG. DMN = default mode network; DST = digit symbol test; MPFC = medial prefrontal cortex; MTG = middle temporal gyrus; NCT = number connecting test; SFG = superior frontal gyrus.

Pearson correlation analysis revealed that the NCT-A scores of all cirrhotic patients negatively correlated the DMN connectivity strength in the MPFC, left SFG, and right MTG. The DST scores positively correlated with the DMN connectivity strength in the right MTG (Figure 3).

### ROC Analysis Results

**FIGURE 4:**
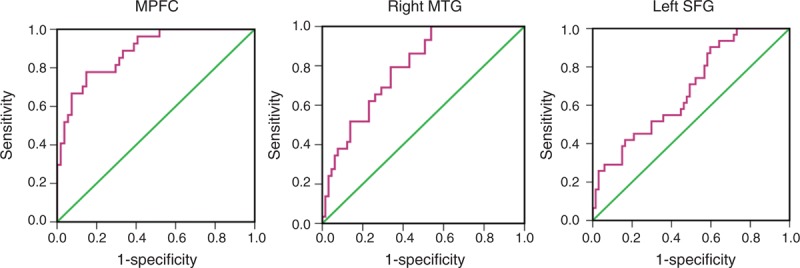
Receiver operator characteristic curve analysis of regional functional connectivity within DMN for diagnosis of minimal hepatic encephalopathy. ROC analyses demonstrate that MPFC, right MTG, and left SFG could differentiate MHE patients from non-HE patients. Among these three regions, MPFC yields optimal value for the early diagnosis of MHE (AUC = 0.884), with a sensitivity of 81.5% and specificity of 70.4%. The right MTG and left SFG have sensitivity of 79.3% and 64.5%, specificity of 64.6% and 52.2%, respectively. AUC = areas under curve of receiver operating characteristics; MHE = minimal hepatic encephalopathy; MPFC = medial prefrontal cortex; MTG = middle temporal gyrus; SFG = superior frontal gyrus.

ROC analysis demonstrated that three regions: the MPFC, right MTG, and left SFG could differentiate MHE patients from non-HE patients (*P* < 0.05) (Figure 4). The areas under curve of ROC, cut-off value, and sensitivity and specificity of each region are summarized in Table 3. Among these three regions, MPFC had the optimal value for the early diagnosis of MHE (AUC = 0.884, cut-off value of connectivity strength = 0.520), with a sensitivity of 81.5% and specificity of 70.4%. The right MTG and left SFG had sensitivity of 79.3% and 64.5%, and specificity of 64.6% and 52.2%, respectively (Table 3).

**TABLE 3:**
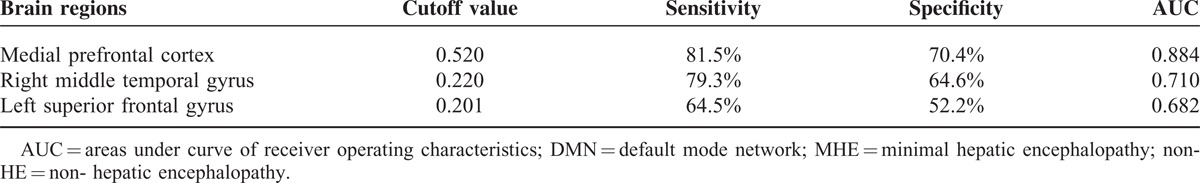
Receiver Operating Characteristics Curves of DMN Functional Connectivity for Discrimination Between MHE and Non-HE

## DISCUSSION

This resting-state fMRI study showed continuous breakdown of DMN functional connectivity from non-HE patients, to MHE patients, which partially correlated with their neuropsychological performances. We also found that the connectivity strength of some DMN regions could differentiate MHE form cirrhotic patients, showing promise as a clinical marker of MHE.

The brain DMN during “rest” is thought to be engaged in the maintenance of the baseline brain activities related to cognitions of self-awareness, episodic memory, and interactive modulation between the internal mind activities and external tasks,^24,27,28^ which is critical to the brain. Several previous studies have investigated the DMN alteration in cirrhotic patients with or without HE. Zhang et al^15^ first reported decreased DMN functional connectivity in a group of HE patients. Another study of the same team demonstrated that MHE patients had altered functional connectivity in some DMN regions without structural alteration, indicating that the DMN functional alteration precedes its structural alterations.^14^ Chen et al^29^ showed a trend of gradually reduced DMN functional connectivity from non-HE patients, to MHE patients using independent component analysis (ICA). Our findings of decreased functional connectivity of DMN in cirrhotic patients are consistent with previous studies. It should be noted that our study has some advantages when compared with previous studies. First, the sample size was large in this study, which would improve the effectiveness of statistical analysis; second, the hypothesis-driven technique with unbiased seed-based functional connectivity of DMN used in this study was more “straight forward” than the ICA, which was often used in previous studies,^30^ and may better characterize the spatial pattern of brain network^24,25^ than the conventional seed-based functional connectivity using a single seed region performed in many previous fMRI literatures; third, there are very few studies have investigated the value of DMN in distinguishing MHE from non-HE.

An important finding in this study is that 3 pivotal nodes of DMN, including the MPFC, left SFG, and right MTG, showed a trend of gradually reduced functional connectivity from healthy controls to non-HE patients and to MHE patients. The MPFC is associated with social cognitive processes, which related to decision making, self regulations, and others.^31,32^ The MTL is engaged in the episodic memory,^33^ and the SFG also participates in the cognitive process.^34^ The reduction of functional connectivity of these DMN regions may underlie the cognitive impairments that are frequently seen in cirrhotic patients, such as impaired attention, and speed of information processing.^1^ Support of our findings can be found in previous studies, in which these aforementioned regions showed decreased cerebral blood flow or metabolism in PET and SPECT,^35–37^ as well as reduced spontaneous brain activity in fMRI.^13,15^

Another important finding is that these 3 above-mentioned DMN regions could differentiate MHE patients from cirrhotic patients, of which the MPFC yielded the highest sensitivity and specificity. The prevalence of MHE in the present study was 33.0%, which is consistent with previous reports.^2,3^ It is reported that most MHE patients would progress to overt HE, which is a severe neuropsychiatric complication of cirrhotic patients, and is associated with poor survival.^10^ Thus, the early diagnosis and treatment of MHE is crucial to prevent the progression of neurological impairment, improve the quality of life and lifespan of cirrhotic patients.^6,38^ The prefrontal cortex is one of the most disturbed regions that have been reported in cirrhotic patients.^39,40^ The present finding that the MPFC could help early diagnosis of MHE objectively and quantitatively provides new insight of prefrontal cortex in the neural underpinning of MHE.

In the present study, we also found significant correlation between the performance of NCT-A, DST, and connectivity strength of some DMN regions. NCT-A tests for psychomotor speed and worse performance is indicated by a longer time for completion. DST tests for psychomotor speed, attention, and visual memory. The number of correctly transcribed symbols indicates performance, that is, a low score means poor performance. The correlation between the DMN functional connectivity and poor neuropsychological performance here is in line with findings in previous neuroimaging studies.^29,14^ Taken together, all these findings suggested that the DMN connectivity has a potential value as an alternative index to characterize the neuropathologic finding in MHE, especially for those who cannot complete the neuropsychological tests (illiteracy or subjects with poor eyesight).

There were some limitations that should be acknowledged in the present study. First, only two neuropsychological tests were used in this study, but these two tests have been recommended to diagnose MHE by the working party of 11th World Congress of Gastroenterology, in the future, we could include broader spectrum of tests to evaluate the cognition function of cirrhotic patients. Second, we did not perform a longitudinal study to assess the changes of DMN function after the therapy of MHE. Further studies are mandatory to address this issue. Third, in the differential diagnosis between MHE and non-HE patients, only ROC analysis was performed in this study. Advanced post-processing algorithms such as the support vector machine (SVM) should be used in the future to investigate the predictive neuroimaging markers for MHE. Fourth, potential effects of medication such as diuretics for controlling ascites in some patients might have an effect on the results of this study. Fifth, exploration of mental activities during rest of participants undergoing fMRI (eg, the resting state questionnaire)^41,42^ is needed to be included in the further studies.

## CONCLUSIONS

In conclusion, we found that cirrhotic patients had gradually reduced DMN functional connectivty from non-HE to MHE. DMN function might be used as a biomarker for early diagnosis of MHE.
